# High Serum Uric Acid Increases the Risk for Nonalcoholic Fatty Liver Disease: A Prospective Observational Study

**DOI:** 10.1371/journal.pone.0011578

**Published:** 2010-07-14

**Authors:** Chengfu Xu, Chaohui Yu, Lei Xu, Min Miao, Youming Li

**Affiliations:** 1 Department of Gastroenterology, the First Affiliated Hospital, College of Medicine, Zhejiang University, Hangzhou, China; 2 Department of Gastroenterology, Ningbo No. 1 Hospital, Ningbo, China; The University of Hong Kong, Hong Kong

## Abstract

Nonalcoholic fatty liver disease (NAFLD) is a common form of chronic liver disease, and serum uric acid is observed to be significantly elevated in NAFLD patients. However, whether this elevation is causal, a bystander, or a consequence of NAFLD remains unclear. We performed a population-based prospective study among the employees of Zhenhai Refining & Chemical Company Ltd., Ningbo, China to investigate whether the elevation of serum uric acid has a casual role for NAFLD. A total of 6890 initially NAFLD-free subjects were followed up for 3 years. Overall, 11.80% (813/6890) subjects developed NAFLD over 3 years of follow-up. The cumulative incidence of NAFLD increased with progressively higher baseline serum uric acid levels (the cumulative incidence was 7.2%, 9.5%, 11.5%, 13.8%, and 17.2% in quintile 1, quintile 2, 3, 4 and 5, respectively; *P* value for trend <0.001). Cox proportional hazards regression analyses showed that serum uric acid levels were independently and positively associated with the risk for incident NAFLD; the age-, gender- and metabolic syndrome adjusted hazard ratio (95% CI) for the subjects in quintile 2, 3, 4 and 5 versus quintile 1 was 1.18 (0.91–1.54), 1.32 (1.03–1.70), 1.39 (1.09–1.78) and 1.50 (1.18–1.92), respectively. Taken together, our prospective observational study showed that elevation of serum uric acid levels independently predicts increase risk for incident NAFLD.

## Introduction

Nonalcoholic fatty liver disease (NAFLD) represents a spectrum of conditions from simple steatosis to nonalcoholic steatohepatitis (NASH) and cirrhosis. It has become one of the most prevalent liver diseases in Western countries, affecting 20%–30% of the general population [1], [2]. NAFLD is an emerging problem in the Asia-Pacific region and the prevalence is likely to increase in the future [3], [4]. Simple steatosis is generally a benign condition; however, NASH can progress to cirrhosis and liver failure [5] and the 5-year survival rate for individuals diagnosed with NASH is estimated to be only 67% [6].

Identifying risk factors is essential for prevention of NAFLD. The development of NAFLD is a multifaceted cascade of physiologic and biochemical events, including genetic [7], environmental [8], metabolic [9], and stress-related factors [10]; the exact risk factors for NAFLD have not been fully clarified. Recent studies showed that NAFLD is closely associated with obesity, hypertension, dyslipidemia, and glucose intolerance, a cluster of metabolic disorders that is now recognized as metabolic syndrome [11], [12]. For this reason, NAFLD has been considered as the hepatic manifestation of metabolic syndrome [12].

Uric acid is the end product of purine metabolism and the serum uric acid (SUA) level is maintained by the balance between uric acid production and excretion [13]. During last decade, an association between elevated SUA levels and metabolic syndrome has been reported [14]–[16]. However, the relationship between NAFLD and SUA has not been clarified. Our recent cross-sectional study demonstrated that SUA levels are significantly elevated in NAFLD patients and that the prevalence rate of NAFLD increases as SUA levels increase [17]. These results suggested that elevated SUA levels may be associated with NAFLD [17]. However, whether this association is causal, a bystander, or a consequence of NAFLD remains under debate.

Therefore, in this study, we performed a population-based prospective study to investigate whether the elevation of SUA has a casual role for NAFLD in the Chinese population.

## Materials and Methods

### Ethics Statement

Verbal informed consent was obtained from each subject before participation in the study after all procedures had been explained. Verbal consent was recorded by the physician who explained the study procedures. Written informed consent was not required because of the observational nature of the investigation. The study protocol and the form of consent were approved by the Ethics Committee of the First Affiliated Hospital, College of Medicine, Zhejiang University.

### Study design and subjects

To identify whether SUA plays a causal role in development of NAFLD, a population-based prospective study was conducted among the employees of Zhenhai Refining & Chemical Company Ltd., Ningbo, China beginning in 2006. The majority of these subjects had taken part in our 2005 cross-sectional study [17].

Certain subjects were excluded at study entry: (i) those diagnosed with fatty liver based on ultrasonography (n = 1795); (ii) those with alcohol consumption greater than 140 g/week for men and 70 g/week for women (n = 596); (iii) those taking antihypertensive or antidiabetic agents, lipid-lowering agents, or hypouricemic agents based on self-reported medical history and medication use (n = 705); (iv) those with a positive history of known liver disease, such as viral hepatitis B or C, or autoimmune hepatitis, or those using hepatotoxic medications (n = 958).

The NAFLD-free cohort thus comprised 7412 subjects (4842 male and 2570 female) with a mean age of 44.4 years. These subjects were classified into various groups according to employee number and scheduled for annual evaluation for 3 years. We excluded 522 subjects (350 male and 172 female, mean age 45.0 years) who did not complete the follow-up examinations. A total of 6890 initially NAFLD-free subjects (4492 male and 2398 female, mean age 44.4 years) were evaluated for the development of NAFLD.

### Baseline examinations

Baseline examinations included a medical history and health habit inventory taken by a physician, anthropometric measurements, hepatic ultrasonic examination, and biochemical measurements. The examinations were administered in the morning. The subjects were instructed to fast for at least 12 hours prior to the examination and to refrain from exercise during the day before their examination.

Blood pressure was measured using an automated sphygmomanometer with the subject in a sitting position. Systolic blood pressure (SBP) and diastolic blood pressure (DBP) were measured at the first and fifth Korotkoff phases, respectively. Standing height and body weight were measured without shoes or outer clothing. Body mass index (BMI, kg/m^2^), used as an index of body fat, was calculated as weight in kilograms divided by height in meters squared. Waist circumference was measured with the measuring tape positioned midway between the lowest rib and the superior border of the iliac crest as the patient exhaled normally [17], [18]. A baseline hepatic ultrasonic examination was also conducted to exclude the subjects with fatty liver or other forms of chronic liver disease.

Biochemical measurements were conducted as our previously described [17]. In brief, fasting whole blood samples were obtained from an antecubital vein and serum samples were separated for the analysis of biochemical values without frozen. The biochemical values included alanine aminotransferase (ALT), aspartate aminotransferase (AST), γ-glutamyltransferase (GGT), triglyceride, total cholesterol, high-density lipoprotein cholesterol (HDL-C), low-density lipoprotein cholesterol (LDL-C), fasting plasma glucose (FPG), creatinine, blood urea nitrogen (BUN), and uric acid. All values were measured by an Olympus AU640 autoanalyzer (Olympus, Kobe, Japan) using standard methods.

### Assessment on outcomes and definitions

The diagnosis of fatty liver was based on the results of abdominal ultrasonography using a Toshiba Nemio 20 sonography machine with a 3.5-MHz probe (Toshiba, Tokyo, Japan). Ultrasound studies were carried out by a trained ultrasonographist who was unaware of the clinical and laboratory data. Hepatic steatosis was diagnosed by characteristic echo patterns according to conventional criteria, such as the evidence of diffuse hyperechogenicity of the liver relative to the kidneys, ultrasound beam attenuation, and poor visualization of intrahepatic structures [19]. NAFLD was diagnosed by abdominal ultrasound following exclusion of alcohol consumption, viral, or autoimmune liver disease [20], [21].

Hyperuricemia was defined as a SUA level>420 µmol/L in men and>360 µmol/L in women [22]. The diagnosis of metabolic syndrome was based on the new International Diabetes Federation definition [23]. For a person to be defined as having the metabolic syndrome they must have: central obesity (defined as waist circumference ≥90 cm for Chinese men and ≥80 cm for Chinese women), plus any two of the following four factors: (i) raised triglyceride level, defined as triglycerides ≥1.7 mmol/L or specific treatment for this lipid abnormality; (ii) reduced HDL-C, defined as HDL-C<1.03 mmol/L in males and <1.29 mmol/L in females; (iii) raised blood pressure, SBP≥130 mmHg or DBP≥85 mmHg, or treatment of previously diagnosed hypertension; (iv) raised FPG, defined as FPG≥5.6 mmol/L, or previously diagnosed type 2 diabetes.

### Statistical analyses

To explore the association between SUA level and risk for incident NAFLD, subjects were stratified according to their baseline SUA levels: quintile 1, ≤295 µmol/L; quintile 2, 296–332 µmol/L; quintile 3, 333–367 µmol/L; quintile 4, 368–409 µmol/L; and quintile 5, ≥410 µmol/L for males; and quintile 1, ≤205 µmol/L; quintile 2, 206–232 µmol/L; quintile 3, 233–262 µmol/L; quintile 4, 263–298 µmol/L; and quintile 5, ≥299 µmol/L for females. The baseline characteristics of the subjects in each quintile were compared. The cumulative incidence of NAFLD was calculated by dividing the number of cases by the numbers of subjects followed up for each SUA quintile.

We applied Cox proportional hazards regression analyses, which are commonly used for risk assessments in prospective studies [24], [25], to estimate hazard ratios for incident NAFLD for each baseline SUA quintile. The subjects within the first quintile were used as reference group. The data were first adjusted for age and gender and then for multiple covariates that might confound the relationship between the SUA and NAFLD. For linear trends of risk, the number of quintiles was used as a continuous variable and tested on each model.

Continuous variables are presented as mean and standard deviation (SD) or medians and interquartile range (IQR), as appropriate. Categorical variables were compared using the χ^2^ test; continuous variables were compared with Mann-Whitney *U* test, student's *t*-test, Kruskal-Wallis *H* test or one-way analysis of variance, depending on the normality of the data. All statistical analyses were performed using the SPSS software package version 11.5 for Windows (SPSS Inc., Chicago, IL). *P*<0.05 (2-tailed) was considered to be statistically significant.

## Results

### Baseline characteristics

At baseline, the mean age (±SD, range) of the 7412 participants was 44.4 years (±13.0, 20–88). Of the 7412 eligible participants, 522 (7.04%) participants did not complete the follow-up examinations. Baseline characteristics, including age, gender, BMI, waist circumference, and blood pressure, and serum liver enzyme, lipid, glucose, creatinine, BUN, and SUA levels were not different between the participants lost to follow-up and those with successful follow-up (Table S1).

A total of 6890 subjects (4492 male and 2398 female) were evaluated yearly over the course of the study. The baseline characteristics of subjects in each SUA quintile are shown in Table 1. Age, BMI, waist circumference, blood pressure, serum liver enzymes (including ALT, AST, and GGT), serum lipids (including TC, TG and LDL-C), FPG, creatinine and BUN all tended to increase at higher SUA levels (all *P*<0.001). In contrast, HDL-C decreased with as SUA increased (*P*<0.001). The gender proportion was not significantly different among the subjects with all five SUA quintiles (*P* = 0.81).

**Table 1:** Baseline characteristics of study subjects according to serum uric acid quintiles.

Variables	All subjects (n = 6890)	Serum uric acid quintile					*P* value^a^
		1 (n = 1383)	2 (n = 1380)	3 (n = 1390)	4 (n = 1397)	5 (n = 1340)	
Age (yr)	44.4 (12.7)	43.7 (12.2)	43.6 (12.5)	43.9 (13.0)	44.1 (13.0)	46.8 (14.1)	<0.001
Gender (male/female, n)	4492/2398	886/497	911/469	903/487	922/475	870/470	0.81
Body mass index (kg/m^2^)	22.40 (2.71)	21.60 (2.56)	22.01 (2.67)	22.29 (2.58)	22.72 (2.66)	23.32 (2.77)	<0.001
Waist circumference (cm)	76.9 (8.2)	74.6 (8.0)	75.8 (7.9)	76.6 (8.2)	77.9 (7.9)	79.6 (7.9)	<0.001
Systolic blood pressure (mmHg)	119.8 (14.8)	117.3 (14.8)	117.9 (14.1)	119.4 (15.2)	121.0 (14.7)	123.6 (15.1)	<0.001
Diastolic blood pressure (mmHg)	75.6 (9.2)	74.2 (9.2)	74.5 (8.9)	75.4 (9.2)	76.3 (9.2)	77.5 (9.1)	<0.001
Alanine aminotransferase (U/L)	21.0 (15.0–29.0)	19.0 (14.0–27.0)	20.0 (15.0–27.0)	21.0 (15.0–29.0)	22.0 (16.0–30.0)	23.0 (17.0–33.0)	<0.001
Aspartate aminotransferase (U/L)	19.0 (16.0–23.0)	18.0 (16.0–21.0)	19.0 (16.0–22.0)	19.0 (16.0–23.0)	19.0 (17.0–23.0)	20.0 (17.0–24.0)	<0.001
γ-Glutamyltransferase (U/L)	17.0 (12.0–26.0)	15.0 (11.0–21.0)	16.0 (12.0–23.0)	17.0 (12.0–25.0)	18.0 (13.0–28.0)	20.0 (14.0–32.0)	<0.001
Triglyceride (mmol/L)	1.15 (0.84–1.63)	0.99 (0.76–1.33)	1.06 (080–1.44)	1.13 (0.84–1.58)	1.25 (0.90–1.77)	1.42 (1.02–2.04)	<0.001
Total cholesterol (mmol/L)	4.76 (0.93)	4.63 (0.89)	4.70 (0.90)	4.74 (0.89)	4.79 (0.95)	4.96 (1.01)	<0.001
HDL cholesterol (mmol/L)	1.29 (1.08–1.57)	1.32 (1.10–1.62)	1.30 (1.08–1.59)	1.28 (1.08–1.58)	1.27 (1.08–1.54)	1.26 (1.08–1.53)	<0.001
LDL cholesterol (mmol/L)	2.68 (0.75)	2.58 (0.73)	2.65 (0.74)	2.68 (0.74)	2.70 (0.77)	2.82 (0.78)	<0.001
Fasting plasma glucose (mmol/L)	4.44 (4.14–4.81)	4.41 (4.13–4.75)	4.43 (4.13–4.78)	4.43 (4.14–4.78)	4.46 (4.15–4.82)	4.52 (4.18–4.94)	<0.001
Creatinine (µmol/L)	72.0 (60.0–81.0)	67.0 (56.0–77.0)	71.0 (59.0–80.0)	72.0 (60.0–80.0)	73.0 (61.0–82.0)	76.0 (65.0–86.0)	<0.001
Blood urea nitrogen (mmol/L)	4.99 (4.21–5.87)	4.74 (3.98–5.53)	4.91 (4.17–5.79)	4.99 (4.20–5.86)	5.06 (4.29–5.93)	5.28 (4.50–6.18)	<0.001

Data are presented as mean (SD) or median (IQR). The subjects were grouped according to quintiles of serum uric acid: quintile 1 (≤295 µmol/L), quintile 2 (296–332 µmol/L), quintile 3 (333–367 µmol/L), quintile 4 (368–409 µmol/L), and quintile 5 (≥410 µmol/L), for male; and quintile 1 (≤205 µmol/L), quintile 2 (206–232 µmol/L), quintile 3 (233–262 µmol/L), quintile 4 (263–298 µmol/L), and quintile 5 (≥299 µmol/L), for female. HDL, high-density lipoprotein; LDL, low-density lipoprotein.

a *P* values are based on χ^2^ test for categorical data and on Kruskal-Wallis *H* test or one-way analysis of variance for continuous data, depending on the normality of the data.

### Association of SUA with incident NAFLD

To explore the association of SUA with incident NAFLD, subjects were stratified into quartiles according to their baseline SUA levels. The cumulative incidence of NAFLD was calculated by dividing the number of cases by the numbers of subjects in each SUA quintile.

**Figure 1:**
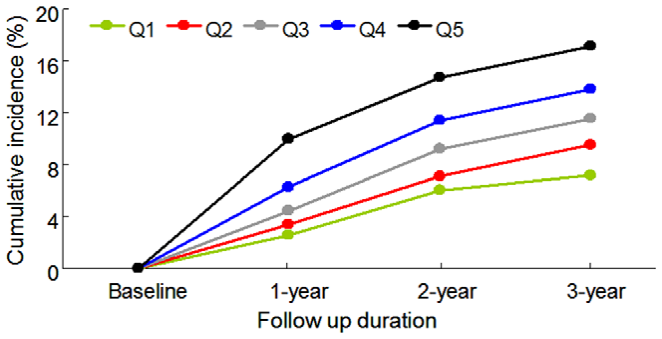
The association between baseline SUA and cumulative incidence of NAFLD. Subjects were stratified into quartiles according to their baseline SUA levels; patients will higher levels of SUA had an increased incidence of NAFLD.

Overall, 813 subjects (605 male and 208 female) developed NAFLD during the 3 year study, corresponding to 13.5% and 8.7% cumulative incidence of NAFLD in men and women, respectively. We observed that baseline SUA quintiles predicted the incidence of NAFLD in a graded and dose-responsive manner (Figure 1). The overall 3-year cumulative incidence of NAFLD was 11.80%, ranging from 7.2% in quintile 1 to 9.5%, 11.5%, 13.8%, and 17.2% in quintile 2, quintile 3, quintile 4, and quintile 5, respectively (*P* for trend<0.001; Figure 1). This tendency also held true for 1-year and 2-year cumulative incidences. The overall 1-year cumulative incidence was 5.3%, ranging from 2.5% in quintile 1 to 9.9% in quintile 5 (*P* for trend<0.001; Figure 1). The overall 2-year cumulative incidence was 9.7%, ranging from 6.0% in quintile 1 to 14.7% in quintile 5 (*P* for trend<0.001; Figure 1). These observations indicated that the subjects with higher baseline SUA levels were more likely to develop NAFLD than those with lower levels.

In contrast to participants without incident NAFLD, those with incident NAFLD were relatively older and predominantly male (Table 2). As expected, baseline BMI, waist circumference, blood pressure, serum liver enzymes, lipids, glucose, renal function and SUA level were significantly different between subjects with and without NAFLD (Table 2).

**Table 2:** Baseline characteristics of study subjects according to follow-up outcomes.

Variables	Subjects developed NAFLD (n = 813)	Subjects did not develop NAFLD (n = 6077)	*t* value	*P* value
Age (yr)	46.0 (12.4)	44.2 (12.8)	3.89	<0.001
Gender (male/female, n)	606/208	3887/2190	34.53^a^	<0.001
Body mass index (kg/m^2^)	23.94 (2.72)	22.20 (2.65)	17.57	<0.001
Waist circumference (cm)	81.8 (8.0)	76.2 (8.0)	18.76	<0.001
Systolic blood pressure (mmHg)	124.5 (14.8)	119.2 (14.7)	9.71	<0.001
Diastolic blood pressure (mmHg)	78.8 (9.1)	75.2 (9.1)	10.53	<0.001
Alanine aminotransferase (U/L)	26.0 (19.0–36.0)	20.0 (15.0–28.0)	12.35 b	<0.001
Aspartate aminotransferase (U/L)	20.0 (17.0–24.0)	19.0 (16.0–23.0)	5.43 b	<0.001
γ-Glutamyltransferase (U/L)	21.0 (15.0–35.0)	16.0 (12.0–24.0)	11.75 b	<0.001
Triglyceride (mmol/L)	1.52 (1.09–2.20)	1.12 (0.82–1.56)	15.23 b	<0.001
Total cholesterol (mmol/L)	4.92 (1.00)	4.74 (0.92)	8.07	<0.001
HDL cholesterol (mmol/L)	1.21 (1.06–1.45)	1.30 (1.09–1.58)	6.05 b	<0.001
LDL cholesterol (mmol/L)	2.80 (0.79)	2.67 (0.75)	4.95	<0.001
Fasting plasma glucose (mmol/L)	4.50 (4.18–4.91)	4.44 (4.14–4.79)	3.58 b	<0.001
Creatinine (µmol/L)	73.0 (62.0–82.0)	71.0 (60.0–81.0)	3.40	0.108
Blood urea nitrogen (mmol/L)	4.99 (4.29–5.92)	4.99 (4.20–5.87)	1.06	0.695
Serum uric acid level (µmol/L)	347.4 (77.1)	314.9 (81.1)	10.80	<0.001

Data are presented as mean (SD) or median (IQR).

a χ^2^ value;

b Z value; HDL, high-density lipoprotein; LDL, low-density lipoprotein.

### High SUA increases the risk of NAFLD

To estimate hazard ratios for incident NAFLD, Cox proportional hazards regression analyses (both univariate and multivariable models) were applied. As shown in Table 3, certain variables were correlated with incident NAFLD in the univariate models: higher SUA level, older age, male gender, higher BMI, higher waist circumference, higher blood pressure, higher serum liver enzymes (including ALT, AST and GGT), higher serum lipids (including TC, TG, and LDL-C), higher FPG, higher creatinine, and lower HDL-C (Table 3). In multivariable models, SUA level was an independent factor related with incident NAFLD (*P* = 0.004; Table 3). BMI, waist circumference, diastolic blood pressure, ALT, AST, TG, HDL-C, and creatinine were also independent factors related with incident NAFLD; whereas age, systolic blood pressure, GGT, TC, LDL-C and FPG were excluded during the multivariable analysis (Table 3).

**Table 3:** Univariable and multivariable Cox Proportional Hazard models of development of NAFLD during 3-year follow-up.

Variables	Mean (SD) or median (IQR)	Univariable models			Multivariable models		
		χ^2^ test	HR (95% CI)	*P* value	χ^2^ test	HR (95% CI)	*P* value
Age (yr)	44.4 (12.7)	13.29	1.01 (1.01–1.02)	<0.001	0.35	1.00 (0.99–1.01)	0.55
Gender (male, n)	4492	29.97	1.55 (1.33–1.82)	<0.001	0.67	1.11 (0.87–1.42)	0.41
Alcohol intake (non-/mild-, n) a	5882/1008	0.361	1.06 (0.88–1.28)	0.55	4.12	0.81 (0.66–0.99)	0.04
Body mass index (kg/m^2^)	22.40 (2.71)	275.54	1.19 (1.17–1.22)	<0.001	11.20	1.07 (1.03–1.11)	0.001
Waist circumference (cm)	76.9 (8.2)	292.47	1.07 (1.07–1.08)	<0.001	26.46	1.04 (1.03–1.06)	<0.001
Systolic blood pressure (mmHg)	119.8 (14.8)	82.65	1.02 (1.01–1.02)	<0.001	0.14	1.00 (0.99–1.01)	0.71
Diastolic blood pressure (mmHg)	75.6 (9.2)	96.32	1.04 (1.03–1.04)	<0.001	5.11	1.01 (1.00–1.02)	0.02
Alanine aminotransferase (U/L)	21.0 (15.0–29.0)	41.39	1.00 (1.00–1.01)	<0.001	4.36	1.01 (1.00–1.01)	0.04
Aspartate aminotransferase (U/L)	19.0 (16.0–23.0)	15.63	1.01 (1.00–1.01)	<0.001	3.33	0.99 (0.97–1.00)	0.07
γ–Glutamyltransferase (U/L)	17.0 (12.0–26.0)	60.82	1.00 (1.00–1.01)	<0.001	2.65	1.00 (1.00–1.00)	0.10
Triglyceride (mmol/L)	1.15 (0.84–1.63)	194.36	1.30 (1.26–1.35)	<0.001	10.8	1.12 (1.05–1.20)	0.001
Total cholesterol (mmol/L)	4.76 (0.93)	21.62	1.18 (1.10–1.27)	<0.001	2.06	1.22 (0.93–1.60)	0.15
HDL cholesterol (mmol/L)	1.29 (1.08–1.57)	29.11	0.58 (0.48–0.71)	<0.001	4.37	0.67 (0.46–0.98)	0.04
LDL cholesterol (mmol/L)	2.68 (0.75)	20.71	1.22 (1.12–1.34)	<0.001	1.51	0.84 (0.64–1.11)	0.22
Fasting plasma glucose (mmol/L)	4.44 (4.14–4.81)	5.88	1.09 (1.02–1.17)	0.02	0.30	0.98 (0.89–1.07)	0.59
Creatinine (µmol/L)	72.0 (60.0–81.0)	3.31	1.00 (1.00–1.00)	0.07	6.22	0.99 (0.99–1.00)	0.01
Blood urea nitrogen (mmol/L)	4.99 (4.21–5.87)	0.77	1.02 (0.97–1.08)	0.38	0.06	1.00 (0.94–1.06)	0.94
Serum uric acid (µmol/L)	318.7 (81.3)	66.65		<0.001	15.78		0.003
Q2 vs Q1			1.33 (1.02–1.72)			1.22 (0.94–1.59)	
Q3 vs Q1			1.61 (1.25–2.07)			1.39 (1.07–1.79)	
Q4 vs Q1			1.93 (1.52–2.46)			1.45 (1.13–1.87)	
Q5 vs Q1			2.40 (1.89–3.04)			1.62 (1.26–2.08)	

a Mild alcohol intake is defined as alcohol consumption less than 140 g/week for men and 70 g/week for women.

CI, confidence interval; HDL, high-density lipoprotein; HR, hazard ratio; IQR, interquartile range; LDL, low-density lipoprotein; Q, quintile; SD, standard deviation.

We also analyzed the hazard ratio of each SUA quartile for incident NAFLD. Baseline SUA levels were positively correlated with the hazard ratios for incident NAFLD, after adjustment for age and gender. In comparison with subjects in quintile 1, the hazard ratios (95% CI) for subjects in quintile 2, quintile 3, quintile 4, and quintile 5 were 1.32 (1.02–1.72), 1.60 (1.25–2.06), 1.92 (1.50–2.44), and 2.34 (1.85–2.97), respectively (*P* for trend <0.001). The relationship of SUA with incident NAFLD remained significant even further adjustment for indictors of metabolic syndrome including waist circumference, systolic blood pressure, diastolic blood pressure, triglyceride, HDL cholesterol and fasting plasma glucose (Table 4). These results demonstrated that SUA level is an independent factor that predicts the development of NAFLD and the risk increases with increase in baseline SUA levels.

**Table 4:** Risk of development of NAFLD according to baseline serum uric acid quintiles in unadjusted and adjusted models.

Models	Quintile of baseline serum uric acid Hazard ratio (95% confidence interval)					χ^2^ test	*P* value
	1 (n = 1383)	2 (n = 1380)	3 (n = 1390)	4 (n = 1397)	5 (n = 1340)		
Unadjusted	1 [reference]	1.33 (1.02–1.72)	1.61 (1.25–2.07)	1.93 (1.52–2.46)	2.40 (1.89–3.04)	66.65	<0.001
Adjusted for age and gender	1 [reference]	1.32 (1.02–1.72)	1.60 (1.25–2.06)	1.92 (1.50–2.44)	2.34 (1.85–2.97)	62.91	<0.001
Adjusted for age, gender and BMI	1 [reference]	1.23 (0.94–1.59)	1.43 (1.11–1.84)	1.60 (1.25–2.04)	1.80 (1.42–2.28)	28.81	<0.001
Adjusted for age, gender and indictors of MS^a^	1 [reference]	1.18 (0.91–1.54)	1.32 (1.03–1.70)	1.39 (1.09–1.78)	1.50 (1.18–1.92)	12.66	0.01
Adjusted for all clinical variables^b^	1 [reference]	1.22 (0.94–1.59)	1.39 (1.07–1.79)	1.45 (1.13–1.87)	1.62 (1.26–2.08)	15.78	0.003

The subjects were grouped according to quintiles of serum uric acid: quintile 1 (≤295 µmol/L), quintile 2 (296–332 µmol/L), quintile 3 (333–367 µmol/L), quintile 4 (368–409 µmol/L), and quintile 5 (≥410 µmol/L), for male; and quintile 1 (≤205 µmol/L), quintile 2 (206–232 µmol/L), quintile 3 (233–262 µmol/L), quintile 4 (263–298 µmol/L), and quintile 5 (≥299 µmol/L), for female. BMI, body mass index; HDL, high-density lipoprotein; LDL, low-density lipoprotein; MS, metabolic syndrome.

a Including age, gender, waist circumference, systolic blood pressure, diastolic blood pressure, triglyceride, HDL cholesterol and fasting plasma glucose.

b Including age, gender, alcohol intake, BMI, waist circumference, systolic blood pressure, diastolic blood pressure, alanine aminotransferase, aspartate aminotransferase, γ-glutamyltransferase, triglyceride, total cholesterol, HDL cholesterol, LDL cholesterol, fasting plasma glucose, creatinine and blood urea nitrogen.

When baseline SUA level was entered as a dichotomous variable, that is, hyperuricemia *vs*. normouricemia, the 3-year cumulative incidence of NAFLD was 17.75% in subjects with hyperuricemia *vs.* 10.98% in subjects with normouricemia (*P*<0.001). In the univariable Cox model, hyperuricemia conferred a hazard ratio of 1.62 (95% CI, 1.35–1.93; *P*<0.001). Adjusting for age, gender, and metabolic syndrome slightly attenuated the hazard ratio value to 1.32 (95% CI, 1.10–1.58; *P* = 0.003). This subgroup analysis further demonstrated that hyperuricemia is an independent risk factor for NAFLD.

## Discussion

Our recent cross-sectional study showed that NAFLD patients had higher SUA levels than healthy controls and that the prevalence of NAFLD was increased at higher SUA levels, suggesting significant association between SUA and NAFLD [17]. However, whether elevated SUA level is a primary cause or a secondary response of NAFLD could not be determined from the cross-sectional study. Therefore, we performed the prospective study described here. We observed that baseline elevation of SUA was positively and significantly associated with increased risk of incident NAFLD in initially NAFLD-free subjects. This association was significant even in the normal range of SUA and was independent of baseline gender, age, metabolic syndrome, and all other clinical variables. These results provide novel evidences for a significant association between SUA and development of NAFLD.

The significant association between SUA and development of NAFLD suggest that high SUA levels may play a causal role in the development of NAFLD. Two potential reasons could explain the mechanism by which high SUA levels participates in the development of NAFLD. The first is that uric acid acts as a strong oxidant in the environment of metabolic syndrome [26]. Recent studies have suggested that elevation of SUA levels is a novel risk factor for the development of metabolic diseases, including hypertension [27], cardiovascular disease [28] and type 2 diabetes mellitus [29]. As NAFLD is a condition closely related to metabolic syndrome, oxidative stress directly caused by uric acid may partially explain why elevation of SUA significant increased the risk of NAFLD. The other explanation is that the generation of uric acid, which is catalyzed by xanthine oxidoreductase, is accompanied by generation of reactive oxygen species. Therefore, increased uric acid generation may result in increased oxidative stress to the liver [30], [31]. Xanthine oxidoreductase-dependent reactive oxygen species might act as the “second hit” that induces NAFLD development [32].

The significant association between SUA and development of NAFLD cannot exclude the possibility that high SUA levels may be just a marker for the development of NAFLD. However, even if this possibility is true, our findings may also have significant clinical implications. Since high SUA levels could predict the development of NAFLD independent of gender, age, metabolic syndrome, and other currently available clinical variables. Furthermore, our recent experimental study observed that hypouricemic therapy consisting of allopurine and benzbromarone, two drugs used clinically to lowering SUA levels, significantly ameliorated hepatic steatosis and decreased serum cholesterol levels in a Mongolian gerbil model of NAFLD [33]. This observation indirectly suggested that SUA may act more than a marker for the development of NAFLD.

The interpretation of this study has several potential limitations. First, the diagnosis of NAFLD was based on ultrasonography, which is not sensitive enough to detect mild steatosis. However, ultrasonography is widely used in epidemiological surveys of NAFLD because it is non-invasive, safe, widely available, portable and sensitivity for detecting hepatic steatosis is acceptable. Sceond, SUA level during follow-up was not included in the analysis. Spearman's rank correlation coefficient was 0.589 (*P*<0.001) for SUA between the baseline examination and the last follow-up examination. This indicates that those who had the higher baseline SUA level tended to be so during follow-up. Therefore, the observed association between elevated baseline SUA and increased risk of incident NAFLD could reflect the effects of SUA over the observed period.

In conclusion, our population-based prospective study clearly demonstrated that SUA is a significant factor associated with the development of NAFLD. Further studies on the precise role of uric acid on the development of NAFLD will enhance our understanding of NAFLD and will eventually guide development of novel therapeutic and prevention strategies for the disease.

## Supporting Information

Table S1 Baseline characteristics of study subjects according to follow-up status.
(0.04 MB DOC)
