# Supplementary material for: High Serum Uric Acid Increases the Risk for Nonalcoholic Fatty Liver Disease: A Prospective Observational Study
Source: PLoS One. 2010 Jul 14;5(7):e11578. doi: 10.1371/journal.pone.0011578 (PMC2904389; doi:10.1371/journal.pone.0011578)
Supplement: Table S1 — Baseline characteristics of study subjects according to follow-up status. (0.04 MB DOC) [file pone.0011578.s001.doc]

**Table S1.** Baseline characteristics of study subjects according to follow-up status

| Variables | Subjects lost to follow-up (n=522) | Subjects successful follow-up (n=6890) | *t* value | *P* value |
| --- | --- | --- | --- | --- |
| Age (yr) | 45.0 (16.5) | 44.4 (12.7) | 1.040 | 0.406 |
| Gender (male/female, n) | 350/172 | 4492/2398 | 0.736^a^ | 0.418 |
| Body mass index (kg/m^2^) | 22.17 (2.64) | 22.40 (2.71) | 1.902 | 0.052 |
| Waist circumference (cm) | 76.7 (8.5) | 76.9 (8.2) | 0.368 | 0.713 |
| Systolic blood pressure (mmHg) | 120.1 (16.9) | 119.8 (14.8) | 0.494 | 0.621 |
| Diastolic blood pressure (mmHg) | 74.5 (9.4) | 75.6 (9.2) | 2.696 | 0.007 |
| Alanine aminotransferase (U/L) | 20.0 (15.0 – 29.0) | 21.0 (15.0 – 29.0) | 1.536^b^ | 0.125 |
| Aspartate aminotransferase (U/L) | 19.0 (16.0 – 23.0) | 19.0 (16.0 – 23.0) | 0.032 ^b^ | 0.974 |
| γ-Glutamyltransferase (U/L) | 17.0 (12.0 – 24.0) | 17.0 (12.0 – 26.0) | 0.099 ^b^ | 0.922 |
| Triglyceride (mmol/L) | 1.11 (0.82 – 1.59) | 1.15 (0.84 – 1.63) | 1.402 ^b^ | 0.161 |
| Total cholesterol (mmol/L) | 4.78 (1.01) | 4.76 (0.93) | 0.252 | 0.801 |
| HDL cholesterol (mmol/L) | 1.29 (1.08 – 1.57) | 1.29 (1.08 – 1.57) | 0.466 ^b^ | 0.641 |
| LDL cholesterol (mmol/L) | 2.71 (0.76) | 2.68 (0.75) | 0.733 | 0.463 |
| Fasting plasma glucose (mmol/L) | 4.43 (4.13 – 4.79) | 4.44 (4.14 – 4.81) | 0.144 ^b^ | 0.885 |
| Creatinine (μmol/L) | 73.0 (60.0 – 83.0) | 72.0 (60.0 – 81.0) | 1.606 | 0.108 |
| Blood urea nitrogen (mmol/L) | 4.49 (4.10 – 5.78) | 4.99 (4.21 – 5.87) | 0.319 | 0.695 |
| Serum uric acid level (μmol/L) | 323.6 (85.5) | 318.7 (81.3) | 1.327 | 0.204 |

Data are presented as mean (SD) or median (IQR).

^a^ χ^2^ value;

^b^ Z value; HDL, high-density lipoprotein; LDL, low-density lipoprotein.
